# Prompt and Appropriate Antimicrobial Therapy Improves Outcomes of NDM-Producing and KPC-Producing *Klebsiella pneumoniae* Bloodstream Infections in Patients Hospitalized for COVID-19: A Comparative Retrospective Case-Series

**DOI:** 10.3390/antibiotics11111519

**Published:** 2022-10-31

**Authors:** Davide Fiore Bavaro, Alessandra Belati, Lucia Diella, Melita Anna Poli, Angela Calamo, Giovanna De Candia, Maurantonio Altamura, Felicia Anna Spadavecchia, Gaetano Brindicci, Nicolò De Gennaro, Francesco Di Gennaro, Annalisa Saracino, Sergio Carbonara

**Affiliations:** 1 Clinic of Infectious Diseases, University of Bari, University Hospital Policlinico, 70121 Bari, Italy; 2 U.O.C. Malattie Infettive, ASL BAT, P.O. V. Emanuele II, 76011 Bisceglie, Italy; 3 UOSVD Patologia Clinica, ASL BAT, P.O. V. Emanuele II, 76011 Bisceglie, Italy

**Keywords:** KPC-*Klebsiella pneumoniae*, NDM-*Klebsiella pneumoniae*, COVID-19, SARS-CoV2, bloodstream infections

## Abstract

Secondary bloodstream infections (BSIs) caused by KPC- and NDM-producing *Klebsiella pneumoniae* (*K.p.*) during the course of COVID-19 infections lead to significant mortality. Herein, a comparative retrospective case series of KPC- or NDM-*K.p.* BSIs occurring in COVID-19 subjects treated with Ceftazidime/Avibactam (CAZ/AVI) for KPC-*K.p.*, or CAZ/AVI+ Aztreonam (ATM) for NDM-K.p is reported. All patients hospitalized for COVID-19 in two Italian hospitals with a BSI between March and September 2021 were included. The main outcome was 14-day mortality. Overall, 44 patients were included: 23 with KPC-*K.p.* and 21 with NDM-*K.p.* BSIs. The median (q1–q3) age was 67 (57–75) years, and 32 (72%) were males. The two groups were similar in terms of baseline comorbidity, or severity of COVID-19. Notably, 14-day mortality of KPC-*K.p.* BSIs and NDM-*K.p.* BSIs (26% vs. 38%, *p* = 0.521) and 28-day mortality (35% vs. 48%, *p* = 0.541) were similar. A Cox regression model of delayed initiation of an appropriate antibiotic therapy after the onset of symptoms independently predicted mortality: initiation between 24 and 72 h (aHR = 12.03; 95% CI = 1.10–130, *p* = 0.041); and initiation after 72h (aHR = 36.9, 95% CI = 3.22–424, *p* = 0.004). Moreover, a trend towards an increased risk of mortality was observed for polymicrobial infections (aHR = 3.73, 95% CI = 0.87–15.8, *p* = 0.074), while a protective effect was observed for a beta-lactam loading dose at the start of treatment (aHR = 0.16, 95% CI = 0.02–1.10, *p* = 0.064). The high mortality of KPC and NDM-*K.p.* BSIs in COVID-19 patients may be reduced by an early and appropriate antibiotic therapy. Further efforts should be made to develop antimicrobial stewardship and infection control programs in COVID-19 wards.

## 1. Introduction

Bloodstream infections (BSIs) are severe diseases burdened by an estimated overall crude mortality rate of 15–30% [1], that may be even higher when caused by nosocomial multidrug-resistant organisms (MDROs) [2] or occurring in “fragile” populations [3,4].

Interestingly, BSIs have been associated with a particularly severe outcome among patients with COVID-19, reaching an estimated overall mortality rate of 30–53% [5,6]. This additional risk of mortality has been associated with multiple factors, including the wide administration of steroids and immunosuppressants, prolonged duration of hospitalization, the use of central venous catheters, and frequent need of intensive care unit (ICU) admission [7,8]. Another important risk factor for mortality is represented by the nosocomial spread of MDROs, a consequence of a dramatic decrease in the application of infection control and prevention procedures caused by the substantial pressure on hospitals during SARS-CoV-2 pandemic waves [9].

In fact, in the COVID-19 setting, outbreaks of carbapenem resistant Enterobacterales (CRE), particularly metallo-β-lactamases producing and serine-carbapenemases producing-*Klebsiella pneumoniae* (*K.p.*), pose a crucial threat to hospitalized patients [10,11,12,13].

Currently, among the few treatment options available, Ceftazidime/Avibactam shows a high efficacy against KPC-*K.p.* in a large multicentric “real life” study [14], whereas the most effective therapies for metallo-β-lactamases producing-*K.p.* are still under investigation. Of note, recent studies suggested a possible role of the association of Aztreonam (ATM) plus CAZ/AVI or Meropenem/Vaborbactam, even if new molecules are under development, thanks to the application of bioinformatic software tools and databases [15,16,17].

Herein, we report the first comparative case series, to our knowledge, of consecutive KPC- and NDM-*K.p.* BSIs occurred in COVID-19 subjects, and their presenting features, antimicrobial therapy approach, and survival outcome are described.

## 2. Materials and Methods

### 2.1. Study Design

This is a retrospective case series collected in two Italian hospitals (Policlinico, Bari and Ospedale “Vittorio Emanuele II,” Bisceglie, Apulia Region, Italy) from 1 March 2021 to 1 September 2021. All data were anonymized and collected in an electronical database. All patients hospitalized for COVID-19 with a BSI were included if they matched the following criteria during the study period: (1) age ≥18 years; and (2) at least one blood culture positive for carbapenem-resistant *K.p.*

All patients were followed-up until 30 days after the BSI episode.

### 2.2. Study Outcome Variables

The main outcome variable was a 14-day mortality, defined as the occurrence of death within 14 days from the index blood culture. Secondary outcome was 28-day mortality.

Moreover, a comparison of clinical characteristics and 14-day mortality between KPC-*K.p.* BSI and NDM-*K.p.* BSI was performed.

### 2.3. Sampling Process

According to current guidelines and hospital protocols, blood cultures were performed for all patients by collecting 20–30 mL of blood per culture set, before starting an empirical antimicrobial therapy. Two bottles (for aerobic and anaerobic bacteria culture, respectively) were collected for each set and immediately placed into a BACT/ALERT^®^ 3D instrument (Biomerieux Inc., Marcy-l’Étoile, France). Positive aerobic blood cultures were subcultured on MacConkey agar, can blood agar, Sabouraud dextrose agar, mannitol-salt agar, and chocolate agar and incubated aerobically at 37 °C for 24 h.

Identification and antibacterial phenotypic susceptibility were tested either on the automated VITEK 2 system or VITEK MS (Biomerieux) according to the manufacturer’s instructions. The interpretative breakpoints of MIC values were based on the criteria of the European committee on antimicrobial susceptibility testing (EUCAST, Version 11.0 2021).

The presence of the bla genes in the carbapenemases, including KPC and NDM, was determined on isolates from the blood cultures by polymerase chain reaction (PCR) using the GeneXpert^®^ System (Cepheid). Whole genome sequencing and electrophoresis were not performed.

### 2.4. Antibiotic Therapy

#### 2.4.1. Empirical and Targeted Therapy

According to the hospitals’ protocols of antimicrobial stewardship, an empirical antibiotic therapy, if indicated, could be prescribed by the physician responsible for the patient, even if this physician does not specialize in Infectious Diseases, whereas the targeted antibiotic regimen must be prescribed by an Infectious Diseases (ID) consultant, based on both the phenotypic and genotypic antimicrobial resistance profile of the CR-*K.p.* blood isolate.

#### 2.4.2. Combination Therapy

The antibiotic treatment was classified as “combination therapy” if other antimicrobials were added to the core regimens represented by Ceftazidime/Avibactam (for KPC-*K.p.* BSIs) or by Ceftazidime/Avibactam plus Aztreonam (in case of NDM-*K.p.* BSIs).

The addition of drugs used for the treatment of other coinfecting bacteria that were not active against CR-*K.p.*, as well as the association of Ceftazidime/Avibactam with Aztreonam for NDM-*K.p.*, were not considered as “combination therapy”.

### 2.5. Data Analysis

Descriptive statistics were produced for demographic, clinical, and laboratory characteristics of patients. Mean and standard deviation (SD) were obtained for normally distributed variables, and median and interquartile range (q1–q3) were obtained for non-normally distributed variables, number, and percentages for categorical variables.

The distribution of outcomes between groups (patients with KPC-*K.p.* BSI versus NDM-*K.p.* BSI, and patients who experienced a 14-day clinical failure versus 14-day clinical success) was analyzed by univariable parametric or nonparametric tests, Kruskal–Wallis or Mann–Whitney U Test (where appropriate) for continuous variables, and with Pearson’s χ2 test (Fisher’s exact test where appropriate) for categorical variables, according to data distribution.

In order to assess predictors of mortality of patients, a univariate Cox regression model was produced; a stepwise multivariable Cox regression was then applied to allow for potential confounders, and was adjusted for variables associated (*p* Value < 0.1) with the endpoint at univariable analysis. Collinear variables were excluded, with no further selection.

Finally, Kaplan–Meier curves estimates were also performed for variables of interest. In all cases, a *p* Value < 0.05 was considered statistically significant. Statistical analysis was performed using STATA “Special Edition” version 16.1 (STATA Corp., Lakeway Drive, TX, USA).

## 3. Results

### 3.1. General Characteristics of the Study Population

**Figure 1:**
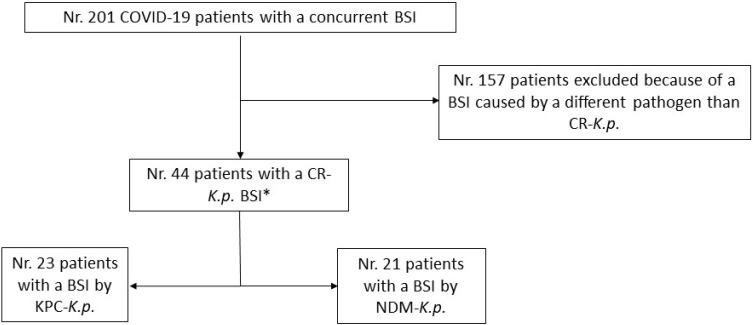
Patient flow-chart. Legend: BSI = Bloodstream infection; CR-*K.p.* = Carbapenem-resistant *K. pneumoniae*; KPC-*K.p.* = *K. pneumoniae* producer of *K. pneumoniae* carbapenemase; NDM-*K.p.* = *K. pneumoniae* producer of New Delhi metallo-β-lactamase. * Twenty-nine out of the 44 patients showed a prior colonization with the same pathogen responsible for the BSI.

Overall, 201 patients hospitalized due to COVID-19, who developed a bacterial BSI in the period of study were enrolled. A carbapenem-resistant *K.p.* was detected by blood cultures as the causative agent of the BSIs in 44 out of 201 subjects, who were included in the study. PCR testing for carbapenemases on the isolates allowed for the identification of the KPC gene (KPC-*K.p.*) in 23 cases (52.3%) and the NDM gene (NDM-*K.p.*) in the remaining 21 (47.7%) (Figure 1).

Table 1 illustrates the characteristics of the study population. At the time of the secondary infection, 32 (73%) patients were males and 12 (27%) were females; the overall median (q1–q3) age was 67 (57–75) years. No significant difference was identified between patients affected by KPC-*K.p.* BSI and those by NDM-*K.p.* BSI in terms of the Charlson comorbidity index (*p* = 0.306), duration of hospitalization before the infection (*p* = 0.769), or COVID-19-related acute respiratory failure at the time of the infection (*p* = 0.800). On the contrary, the status of colonization before the BSI, and the ward of symptoms onset significantly differed between the two patient groups (*p* = 0.044 and 0.001, respectively).

### 3.2. Characteristics of BSIs

Table 2 shows the characteristics of BSIs and data regarding the antibiotic therapies administered.

Overall, a significant difference in the source of BSIs was found between the KPC-*K.p.* and NDM-*K.p.* group (*p* = 0.013); indeed, the most frequent source of infections caused by NDM-*K.p.* was the urinary tract (48%), while KPC-*K.p.* BSIs were associated most frequently with an infected central venous catheter (CVC) (48%).

Notably, 10 cases of polymicrobial BSIs were documented (5 cases in each group), including 5 patients with a coinfection by carbapenem-resistant Acinetobacter baumannii. Moreover, two coinfecting pathogens (other than *K.p.*) were involved in each of four cases.

Finally, no difference between the two groups was found in terms of septic shock at presentation (43% vs. 38% for KPC-*K.p.* and NDM-*K.p.*, respectively; *p* = 0.707).

### 3.3. Antibiotic Therapy

As illustrated in Table 2, all patients with a BSI caused by KPC-*K.p.* were treated with CAZ/AVI (2.5 g every 8 h infused over 3 h), and all BSIs determined by NDM-*K.p.* were administered CAZ/AVI (same dose as in the KPC-*K.p.* group) plus ATM (2 g every 8 h infused over 3 h). No KPC-*K.p.* showed in vitro resistance to CAZ/AVI. In cases of renal failure, the dosages of both CAZ/AVI an ATM were adapted accordingly.

CAZ/AVI was used in combination with other antibiotics in 70% of KPC-*K.p.* BSIs (70%); conversely, in NDM-*K.p.* infections, CAZ/AVI + ATM were associated with other drugs only in 14% (3/21) of cases (*p* < 0.001), because of the presence of a co-infecting pathogen resistant to both drugs (carbapenem-resistant A. baumannii in two cases and P.aeruginosa in one case).

Doses of companion antimicrobials used in the absence of renal failure were prescribed according to hospital protocols of antimicrobial stewardship as follows: (i) Meropenem: 2 g every 8 h infused over four hours; (ii) Colistin: loading dose of 9 million UI followed by 4, 5 million UI every 12 h infused over 60–90 min; (iii) Fosfomycin: 4 gr every 6 h or 6 gr every 8 h infused over 2 h; (iv) Tigecycline: loading dose of 200 mg followed by 100 mg bid; (v) Gentamicin: 5 mg/Kg as a single daily dose (infused over 60 min); and (vi) Amikacin: 15–20 mg/Kg as a single daily dose (infused over 60 min).

In patients with renal failure, doses of antimicrobials were adjusted accordingly.

Notably, beta-lactams regimens were initiated with a loading dose in 41% of patients (57% vs. 24%, of KPC-*K.p.* vs. NDM-*K.p.*, respectively, *p* = 0.036).

Of note, CAZ/AVI (or CAZ/AVI + ATM in NDM-*K.p.* BSIs) was administered in 45% of patients within 24 h from the onset of symptoms; in 32% between 24 and 72 h; and in the remaining 23% later than 72 h from the onset of BSI-related clinical manifestations.

Of note, no further antimicrobial therapies were prescribed to patients during the study period.

### 3.4. Risk of Mortality

The 14-day mortality and the 28-day mortality were similar in BSIs caused by KPC-*K.p.* and in those by NDM-*K.p.* (26% vs. 38%, *p* = 0.521, and 35% vs. 48%, *p* = 0.541, respectively) (Table 2).

On the contrary, as depicted in Table 3, the 14-day mortality was significantly associated with critical patient conditions represented by either the need for mechanical ventilation (*p* = 0.013) or the occurrence of septic shock (*p* = 0.049).

The time between symptom onset and the initiation of an adequate antibiotic therapy was also associated with the 14-day mortality (*p* < 0.001): within 24 h (7% mortality), between 24 and 72 h (33%), and more than 72 h (64%).

By performing a univariate and a stepwise multivariate Cox regression model (Table 4) adjusted for multiple covariates, the only independent predictor of 14-day mortality was a delayed initiation of an appropriate antibiotic therapy from the onset of clinical manifestations related to the BSI: initiation between 24 and 72 h [adjusted hazard ratio (aHR) = 12.03; 95% confidence interval (95% CI) = 1.10–130, *p* = 0.041], and initiation after 72h (aHR = 36.9, 95% CI = 3.22–424, *p* = 0.004) (reference: initiation within 24 h from symptom onset aHR = 1).

**Figure 2:**
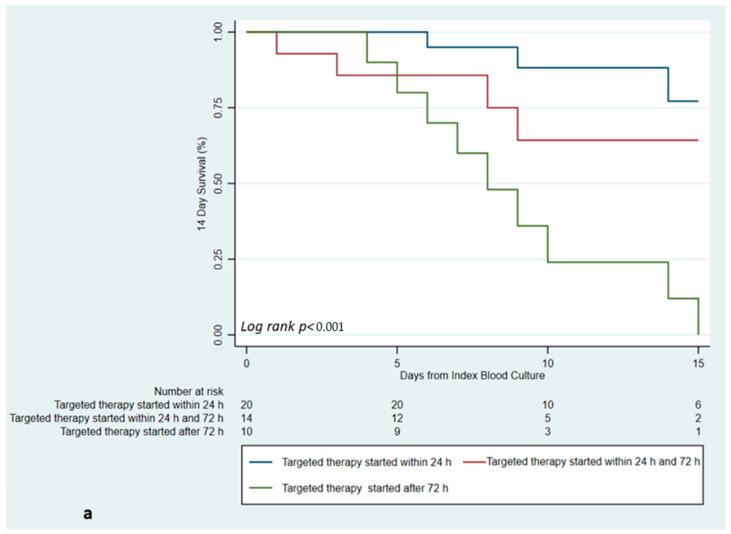

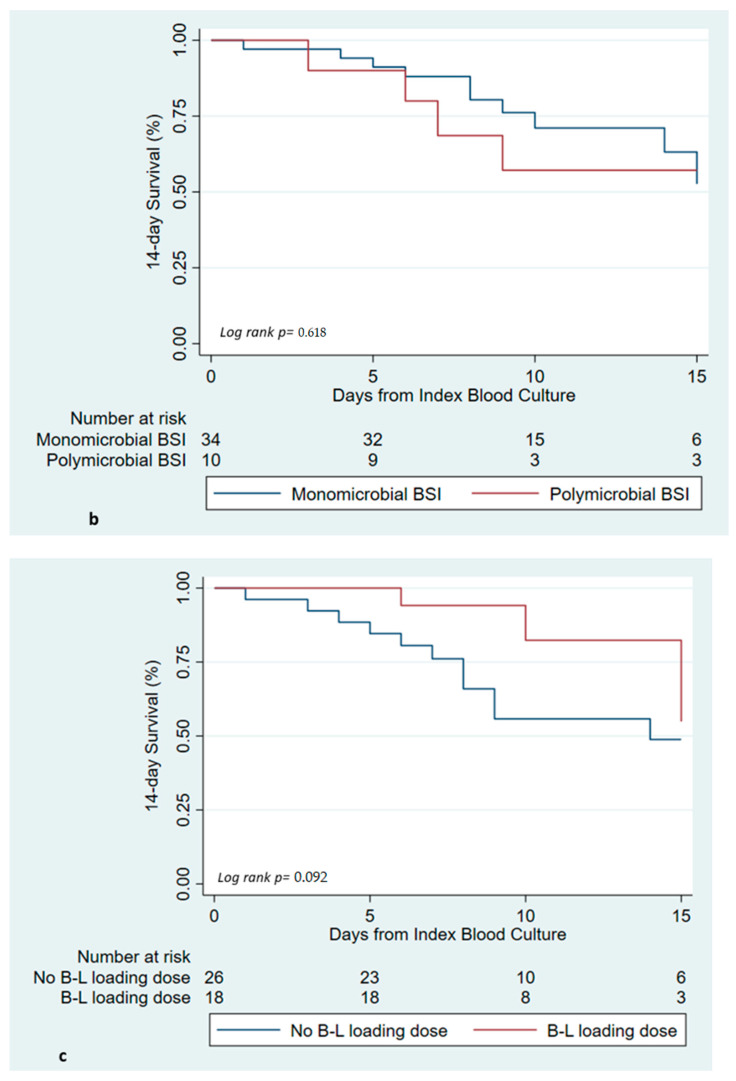
Kaplan–Meier survival curves for 14-day risk of mortality according to (**a**) time from symptom onset to targeted therapy; (**b**) mono- or polymicrobial BSI; (**c**) use of Beta-lactam loading dose.

Interestingly, a trend towards an increased risk of mortality was also observed for polymicrobial infections (aHR = 3.73, 95% CI = 0.87–15.8, *p* = 0.074), while a trend towards a protective effect was observed for the use of a loading dose at the start of beta-lactam therapy (aHR = 0.16, 95% CI = 0.02–1.10, *p* = 0.064). Both these statistical associations were explored by performing Kaplan–Meier survival curves: the analysis confirmed the significant association between delayed treatment initiation and mortality (Figure 2a, *p* < 0.001). Conversely, no significant association was observed with polymicrobial infection (Figure 2b, *p* = 0.618), while a trend toward a protective effect was observed for the administration of a beta-lactam loading dose (Figure 2c, *p* = 0.092).

## 4. Discussion

Previous studies describe characteristics of CR-*K.p.* infection in COVID-19 patients and find that COVID-19 patients [18] have a higher risk of CR-*K.p.* infections, leading to higher mortality than in non-COVID-19 patients. These studies also examine different characteristics and outcomes of several pathotypes of CR-*K.p.* in southern Italy [19], but to the best of our knowledge, this is the first study comparing clinical characteristics and outcomes of KPC- and NDM-*K.p.* BSIs occurring in subjects hospitalized for COVID-19. Overall, patients of both groups had similar presenting conditions, including the degree of COVID-19 severity (need for low-middle oxygen flux therapy, non-invasive ventilation, or mechanical ventilation), as well as the distribution of comorbidities between those who presented with KPC- and NDM-*K.p.* BSIs. Moreover, in this study, no specific association between different outcomes and gender was noticed; accordingly, no further analysis was performed in this regard. However, it should be noted that the number of female patients was quite low, which hampers the statistical power of potential post hoc analysis.

Notably, in both groups, the BSIs by CRE were heralded by a prior colonization by the same MDRO in the majority of patients; this finding suggests the need for a close clinical monitoring of hospitalized patients with COVID-19 and colonization by a CR-*K.p.* in order to both promptly recognize the switch from a condition of mere colonization to infection, and initiate an appropriate therapy in a timely manner. In fact, BSIs represent a medical urgency, and in our study population, the time from the clinical onset to the initiation of an effective antimicrobial therapy represented the only independent variable for mortality (14-day mortality = 7% if treatment started within 24 h, 29% if between 24 and 72 h, and 64% if later than 72). This correlation was consistent with similar findings in previously published studies among patients affected by severe infections caused by MDRO [20], although this association was not explored previously in patients with COVID-19.

The need for an early appropriate treatment of BSIs caused by CR-*K.p.* implies that such a therapy has often to be initiated empirically soon after the clinical onset of bacteremia, before results of blood-cultures are ready. In these circumstances, the choice of molecules should be driven by risk factors for CRE infections reported by the literature [6], including a known colonization by these pathogens [21], as well as by both the hospital- and ward-based prevalence of CRE. These criteria for an effective empirical regimen require, in turn, several interventions, including the following: an adequate screening for CRE colonization at the time of admission of patients with risk factors for CRE or in wards at risk for the same pathogens, a prompt recognition of clinical onset of BSIs along with a timely collection of blood cultures, and the gene identification of carbapenemases produced in patients either colonized or infected by CRE.

Overall, BSIs by CR-*K.p.* occurred in all patients a median of 21 days (IQR 11–38 days) after hospitalization, without significative differences between KPC- or NDM-*K. pneumoniae* as the causative agent. Different studies reported molecular pathways of resistance in both NDM- and KPC-*K.p.*, which make *K.p.* particularly invasive, aggressive, and capable of causing hospital outbreaks. [22,23].

Moreover, the frequency of polymicrobial infections has represented a further remarkable finding of our study. In fact, we detected a coinfection by “difficult-to-treat” pathogens other than CR-*K.p.* in one out of four patients. Taken together, these findings highlight both the issue of secondary healthcare associated bacterial infections in patients hospitalized with COVID-19, which have been associated in previous investigations with the duration of hospital stay [24], as well as the need for implemented infection control measures in hospital wards dedicated to COVID-19 patients.

Notably, in our cohort, the 14-day and 28-day mortality rates among the NDM-*K.p.* and KPC-K.p BSIs resulted similarly, as opposed to findings in previous reports [21,25]. This finding may be explainable through multiple observations. At first, in our case series, all patients were treated with new beta-lactams/beta-lactams inhibitors (CAZ/AVI for the KPC-*K.p.* and CAZ/AVI plus ATM for NDM K.p) and not with more common combination regimens based mainly on Colistin, Aminoglycosides, and Tigecycline, which showed to be less effective when compared with new drugs on “difficult to treat” Gram negative bacteria [15,26,27,28]. Second, it should be considered that both a beta-lactams loading dose and the co-administration of other antibiotics to the core therapy (CAZ/AVI for the KPC-*K.p.* and CAZ/AVI plus ATM for NDM K.p) occurred significantly more frequently in the KPC-*K.p.* group than in the NDM-*K.p.* group. These two approaches of therapy intensification might have limited the mortality rate in the latter group to some extent.

In fact, in the multivariate analysis, a protective trend for the 14-day survival was observed for beta-lactam loading dose utilization, consistent with similar findings of current literature [29,30,31]. Conversely, we found no correlation between mortality and a combination therapy, for which utility in the management of these difficult-to-treat infections remains, therefore, questionable, at least with regard to the new agents herein reported [14]. However, neither our data nor those already published thus far provide enough evidence that a combination therapy does not reduce at least the risk of resistance selection to the administered drugs.

Overall, this study has some limitations, including the retrospective approach, the short time span of the study, and the low number of patients involved, which did not allow for further analysis on subgroups of patients to explore other variables associated with the outcome of the study. Moreover, in this study, no specific association between different outcomes and gender was noticed; accordingly, no deeper analysis was performed in this regard. However, it should be noted that the number of female patients was quite low, hampering the statistical power of potential post hoc analysis. In addition, we acknowledge that in this study a comparison with patients not infected by SARS-CoV-2 is lacking; future studies evaluating this aspect could be useful to provide meaningful results related to COVID-19 itself, and its impact on BSI severity.

Still, it should be noticed that the characteristics of this study allow for very uniform data recording and the inclusion of a homogenous cohort as demonstrated by the similarity of basal conditions of the subjects involved.

Moreover, among other limitations, we acknowledge the absence of therapeutic drug monitoring of beta-lactams to better assess implications of loading doses and extended infusion, and the absence of synergy testing between CAZ-AVI and ATM on NDM-*K.p.* isolates.

## 5. Conclusions

This is the first study comparing outcomes of KPC- and NDM-*K.p.* BSI in COVID-19 patients. This infection represents a threatening health-care related complication of hospitalized patients with COVID-19, regardless of severity or respiratory failure. In this context, a prompt treatment with CAZ-AVI for KPC-*K.p.* BSIs and CAZ-AVI + ATM for NDM-*K.p.* BSIs could yield a similar survival outcome and reduce mortality. Similarly, a beta-lactams loading dose may be a useful intensification strategy.

## Figures and Tables

**Table 1:** General characteristics of the study population.

Characteristic *	Overall (n. 44)	BSIs Caused by KPC-*K.p*. (n. 23)	BSIs Caused by NDM-*K.p.* (n. 21)	*p* Value
Age (years), median (q1–q3)	67 (57–75)	62 (52–75)	70 (65–79)	0.176
Male Sex, n. (%)	32 (73)	15 (65)	17 (81)	0.318
Female Sex, n. (%)	12 (27)	8 (35)	4 (19)	
Charlson Comorbidity Index, median (q1–q3)	4 (2–7)	4 (2–9)	3 (2–6)	0.306
Comorbidity, n (%)				
*Hypertension*	11 (25)	7 (30)	4 (19)	0.494
*Chronic Obstructive Pulmonary Disease*	10 (23)	6 (26)	4 (19)	0.724
*Type II Diabetes*	14 (32)	8 (35)	6 (29)	0.752
*Chronic Kidney Diseases*	14 (32)	8 (35)	6 (29)	0.752
*Dialysis*	5 (11)	5 (22)	0	0.050
*Obesity*	14 (32)	7 (30)	7 (33)	0.999
*Malignancies*	5 (11)	4 (17)	1 (5)	0.348
COVID-19-related Acute Respiratory Failure at the time of infection, n (%)				
*Low flux oxygen therapy*	11 (25)	5 (22)	6 (29)	
*Non-invasive Ventilation*	19 (43)	11 (48)	8 (38)	0.800
*Mechanical Ventilation*	14 (32)	8 (35)	7 (33)	
Hospital ward of evaluation, n. (%)				
*Infectious Diseases/General Medicine*	16 (36)	3 (13)	13 (62)	0.001 §
*Sub-Intensive/Intermediate Care Units*	13 (30)	11 (48)	2 (10)	
*Intensive Care Units*	15 (34)	9 (39)	6 (29)	
CR-*K.p*. colonization before infection, n. (%)	29 (66)	12 (52)	17 (81)	0.044
Duration of Hospitalization before diagnosis of infection (days), median (q1–q3)	21 (11–38)	17 (11–38)	23 (11–38)	0.769

**Legend:** BSI = bloodstream infection; *K.p.* = *Klebsiella pneumoniae*; CR = Carbapenem resistant; CRE = carbapenem resistant Enterobacteriaceae. Boldface = statistically significant (*p* < 0.05); (q1–q3) = Interquartile range. (*) all variables are expressed as number (%) of patients, unless otherwise specified. *Post-hoc analysis* (§) Infectious Diseases/General Medicine (*p* < 0.001); Sub-Intensive/Intermediate Care Units (*p* < 0.001); Intensive Care Units (*p* = 0.460).

**Table 2:** Characteristics of BSIs and antibiotic therapies prescribed.

Characteristic *	Overall (n. 44)	BSIs Caused by KPC-*K.p.* (n. 23)	BSIs Caused by NDM-*K.p.* (n.21)	*p* Value
Source of bloodstream infection, n (%)				
*Urinary Tract*	13 (30)	3 (13)	10 (48)	
*Primary Bacteriemia/Unknown Source*	15 (34)	7 (30)	8 (38)	0.013 ^§^
*CVC-related bacteriemia*	14 (32)	11 (48)	3 (14)	
*Ventilator-associated pneumonia*	2 (5)	2 (9)	0	
Septic Shock at presentation, n (%)	18 (41)	10 (43)	8 (38)	0.707
Polymicrobial infections, n (%)	10 (23)	5 (22)	5 (24)	0.999
Other pathogen(s) than *K.p.*, n (%):				
*CR-A. baumannii*	6 (60)	4 (80)	2 (40)	n.e.
*CR-A. baumannii (only)*	*3 (50)*	*1 (25)*	*2 (40)*	
*CR-A. baumannii + Enterococcus* spp.	*1 (16)*	*1 (25)*	*0*	
*CR-A. baumannii + S. marcescens*	*1 (16)*	*1 (25)*	*0*	
*CR-A. baumannii + C. glabrata*	*1 (16)*	*1 (25)*	*0*	
*Enterococcus* spp.	*3 (30)*	*1 (20)*	*2 (40)*	
*Enterococcus* spp. *+ P. aeruginosa*	1 (10)	0	1 (20)	
Time to appropriate antimicrobial therapy, n (%)				
*within 24 h*	20 (45)	12 (52)	8 (38)	
*between 24 and 72 h*	14 (32)	7 (30)	7 (33)	0.587
*>72 h*	10 (23)	4 (17)	6 (29)	
Beta-lactams loading dose, n (%)	18 (41)	13 (57)	5 (24)	0.036
Antibiotic therapy for *K. pneumoniae* BSI, n (%)				
*Ceftazidime-Avibactam*	23 (52)	23 (100)	0	<0.001
*Ceftazidime-Avibactam + Aztreonam*	21 (48)	0	21 (100)	
Companion antibiotics added to therapy for BSI, n (%)	19 (43)	16 (70)	3 (14)	<0.001
*Colistin*	3 (16)	1 (6)	2 (67)	n.e.
*Aminoglycosides*	6 (32)	6 (38)	0	
*Fosfomycin*	3 (16)	3 (19)	0	
*Colistin + Tigecycline*	2 (11)	1 (6)	1 (33)	
*Meropenem*	5 (26)	5 (31)	0	
14-day Mortality, n (%)	14 (32)	6 (26)	8 (38)	0.521
28-day Mortality, n (%)	18 (41)	8 (35)	10 (48)	0.541

**Legend:** BSI = bloodstream infection. *K.p. = Klebsiella pneumoniae.* CR = carbapenem resistant; n.e. = not evaluable. (*) all variables are expressed as number (%) of patients. *Post-hoc analysis.* (§) Urinary Tract (*p* = 0.012); Primary Bacteriemia/Unknown Source (*p* = 0.592); CVC-related bacteriemia (*p* = 0.017); Ventilator-associated pneumonia (*p* = 0.510).

**Table 3:** Analysis of determinants of 14-day mortality.

Variable *	Overall (n. 44)	14-Day Mortality		*p* Value
		Deaths (n.14)	Survivors (n.30)	
Age (years), median (q1–q3)	67 (57–75)	72 (62–75)	65 (56–75)	0.284
Male Sex, n (%)	32 (73)	9 (64)	23 (77)	0.475
Female Sex, n (%)	12 (27)	5 (36)	7 (23)	
Charlson Comorbidity Index, median (q1–q3)	4 (2–7)	4 (3–7)	3 (2–6)	0.397
Degree of Acute Respiratory Failure at the time of infection				
*Low flux oxygen therapy*	11 (25)	3 (21)	8 (27)	0.055 ^§^
*Non-invasive Ventilation*	19 (43)	3 (21)	16 (53)	
*Mechanical Ventilation*	14 (32)	8 (57)	6 (20)	
Septic Shock at presentation, n (%)	18 (41)	9 (64)	9 (30)	0.049
Source of bloodstream infection, n (%)				
*Urinary Tract*	13 (30)	1 (7)	12 (40)	0.093 ^§§^
*Primary Bacteriemia/Unknown Source*	15 (34)	7 (50)	8 (27)	
*CVC-related bacteriemia*	14 (32)	5 (36)	9 (30)	
*Ventilator-associated pneumonia*	2 (5)	1 (7)	1 (3)	
Type of carbapenemases produced by *K. pneumoniae*				
*KPC*	23 (52)	6 (43)	17 (57)	
*NDM*	21 (48)	8 (57)	13 (43)	0.521
Time to appropriate antimicrobial therapy, n (%)				
*within 24 h*	20 (45)	1 (7)	19 (63)	
*between 24 and 72 h*	14 (32)	4 (29)	10 (33)	<0.001 ^#^
*>72 h*	10 (23)	9 (64)	1 (3)	
Loading dose of targeted antimicrobial therapy, n (%)	18 (41)	3 (21)	15 (50)	0.104
Combination therapy, n (%)	19 (43)	7 (50)	12 (40)	0.745
Polymicrobial BSI, n (%)	10 (23)	4 (29)	6 (20)	0.701

**Legend:** BSI = bloodstream infection. CR-*K-p-* = Carbapenem-resistant *Klebsiella pneumoniae.* (*) all variables are expressed as number (n) of patients. *Post-hoc analysis.* (§) Low flux oxygen therapy (*p* = 0.708); Non-invasive Ventilation (*p* = 0.046); Mechanical Ventilation (*p* = 0.013); (§§) Urinary Tract (*p* = 0.026); Primary Bacteriemia/Unknown Source (*p* = 0.128); CVC-related bacteriemia (*p* = 0.704); Ventilator-associated pneumonia (*p* = 0.572); (#) within 24 h (*p* < 0.001); between 24 and 72 h (*p* = 0.751); >72 h (*p* < 0.001).

**Table 4:** Cox regression model for the risk of 14-day mortality.

	Univariate Analysis			Multivariate Analysis		
	HR	95% CI	*p* Value	aHR	95% CI	*p* Value
Age, per 1 year increase	1.01	0.97–1.06	0.431	1.03	0.93–1.13	0.520
Male Sex	0.70	0.23–2.12	0.538	0.39	0.64–2.37	0.308
Charlson Comorbidity Index, per 1 point increase	1.04	0.87–1.24	0.624	0.97	0.67–1.40	0.909
Degree of Acute Respiratory Failure at the time of infection						
*Low flux oxygen therapy*	1			\		
*Non-invasive Ventilation*	0.67	0.13–3.37	0.636	\		
*Mechanical Ventilation*	2.39	0.63–9.02	0.198	\		
Septic Shock at presentation, n (%)	2.59	0.86–7.77	0.088	1.38	0.33–5.72	0.656
Source of bloodstream infection, n (%)						
*Urinary Tract*	1			1		
*Source other than Urinary Tract*	4.66	0.60–35.8	0.139	4.81	0.32–71.4	0.254
Carbapenemases produced by *K. pneumoniae*						
*KPC*	1			1		
*NDM*	1.89	0.64–5.55	0.242	0.92	0.13–6.53	0.939
Time to appropriate antimicrobial therapy, n (%)						
*within 24 h*	1			1		
*between 24 and 72 h*	8.35	0.92–75	0.058	12.03	1.10–130	0.041
*>72 h*	26.83	3.37–213	0.002	36.9	3.22–424	0.004
Loading dose of Beta-lactams, n (%)	0.35	0.09–1.26	0.110	0.16	0.02–1.10	0.064
Combination therapy, n (%)	1.22	0.42–3.49	0.707	\		
Polymicrobial/ multiple infections, n (%)	1.34	0.41–4.29	0.622	3.73	0.87–15.8	0.074

## Data Availability

The dataset used for this study can be obtained from the corresponding author on reasonable request.

## References

[1] Ammerlaan H.S., Harbarth S., Buiting A.G., Crook D.W., Fitzpatrick F., Hanberger H., Herwaldt L.A., van Keulen P.H.J., Kluytmans J.A.J.W., Kola A. (2013). Secular trends in nosocomial bloodstream infections: Antibiotic-resistant bacteria increase the total burden of infection. Clin. Infect. Dis..

[2] Nagao M. (2013). A multicentre analysis of epidemiology of the nosocomial bloodstream infections in Japanese university hospitals. Clin. Microbiol. Infect..

[3] Goto M., Al-Hasan M.N. (2013). Overall burden of bloodstream infection and nosocomial bloodstream infection in North America and Europe. Clin. Microbiol. Infect..

[4] Bavaro D.F., Pizzutilo P., Catino A., Signorile F., Pesola F., Di Gennaro F., Cassiano S., Marech I., Lamorgese V., Angarano G. (2021). Incidence of Infections and Predictors of Mortality During Checkpoint Inhibitor Immunotherapy in Patients with Advanced Lung Cancer: A Retrospective Cohort Study. Open Forum Infect. Dis..

[5] Ippolito M., Simone B., Filisina C., Catalanotto F.R., Catalisano G., Marino C., Misseri G., Giarratano A., Cortegiani A. (2021). Bloodstream Infections in Hospitalized Patients with COVID-19: A Systematic Review and Meta-Analysis. Microorganisms.

[6] Segala F.V., Bavaro D.F., Di Gennaro F., Salvati F., Marotta C., Saracino A., Murri R., Fantoni M. (2021). Impact of SARS-CoV-2 Epidemic on Antimicrobial Resistance: A Literature Review. Viruses.

[7] Khatri A., Malhotra P., Izard S., Kim A., Oppenheim M., Gautam-Goyal P., Chen T., Doan T.L., Berlinrut I., Niknam N. (2021). Hospital-Acquired Bloodstream Infections in Patients Hospitalized with Severe Acute Respiratory Syndrome Coronavirus 2 Infection (Coronavirus Disease 2019): Association with Immunosuppressive Therapies. Open Forum Infect. Dis..

[8] Balena F., Bavaro D.F., Fabrizio C., Bottalico I.F., Calamo A., Santoro C.R., Brindicci G., Bruno G., Mastroianni A., Greco S. (2020). Tocilizumab and corticosteroids for COVID-19 treatment in elderly patients. Gerontol. Geriatr..

[9] Weiner-Lastinger L.M., Pattabiraman V., Konnor R.Y., Patel P.R., Wong E., Xu S.Y., Smith B., Edwards J.R., Dudeck M.A. (2022). The impact of coronavirus disease 2019 (COVID-19) on healthcare-associated infections in 2020: A summary of data reported to the National Healthcare Safety Network. Infect. Control Hosp. Epidemiol..

[10] Karruli A., Bocciam F., Gagliardi M., Patauner F., Ursi M.P., Sommese P., De Rosa R., Murino P., Ruocco G., Corcione A. (2021). Multidrug-Resistant Infections and Outcome of Critically Ill Patients with Coronavirus Disease 2019: A Single Center Experience. Microb. Drug Resist..

[11] Papadimitriou-Olivgeris M., Bartzavali C., Lambropoulou A., Solomou A., Tsiata E., Anastassiou E.D., Fligou F., Marangos M., Spiliopoulou I., Christofidou M. (2019). Reversal of carbapenemase-producing *Klebsiella pneumoniae* epidemiology from blaKPC- to blaVIM-harbouring isolates in a Greek ICU after introduction of ceftazidime/avibactam. J. Antimicrob. Chemother..

[12] Nori P., Szymczak W., Puius Y., Sharma A., Cowman K., Gialanella P., Fleischner Z., Corpuz M., Torres-Isasiga J., Bartash R. (2020). Emerging Co-Pathogens: New Delhi Metallo-beta-lactamase producing Enterobacterales Infections in New York City COVID-19 Patients. Int. J. Antimicrob. Agents.

[13] Falcone M., Suardi L.R., Tiseo G., Galfo V., Occhineri S., Verdenelli S., Ceccarelli G., Poli M., Merli M., Bavaro D.F. (2022). Superinfections caused by carbapenem-resistant Enterobacterales in hospitalized patients with COVID-19: A multicentre observational study from Italy (CREVID Study). JAC Antimicrob. Resist..

[14] Tumbarello M., Raffaelli F., Giannella M., Mantengoli E., Mularoni A., Venditti M., De Rosa F.G., Sarmati L., Bassetti M., Brindicci G. (2021). Ceftazidime-avibactam use for KPC-*K.p.* infections: A retrospective observational multicenter study. Clin. Infect. Dis..

[15] Falcone M., Daikos G.L., Tiseo G., Bassoulis D., Giordano C., Galfo V., Leonildi A., Tagliaferri E., Barnini S., Sani S. (2021). Efficacy of Ceftazidime-avibactam Plus Aztreonam in Patients with Bloodstream Infections Caused by Metallo-β-lactamase-Producing Enterobacterales. Clin. Infect. Dis..

[16] Belati A., Bavaro D.F., Diella L., De Gennaro N., Di Gennaro F., Saracino A. (2022). Meropenem/Vaborbactam Plus Aztreonam as a Possible Treatment Strategy for Bloodstream Infections Caused by Ceftazidime/Avibactam-Resistant *Klebsiella pneumoniae*: A Retrospective Case Series and Literature Review. Antibiotics.

[17] Behzadi P., García-Perdomo H.A., Karpiński T.M., Issakhanian L. (2020). Metallo-ß-lactamases: A review. Mol. Biol. Rep..

[18] Pintado V., Ruiz-Garbajosa P., Escudero-Sanchez R., Gioia F., Herrera S., Vizcarra P., Fortún J., Cobo J., Martín-Dávila P., Morosini M.I. (2022). Carbapenemase-producing Enterobacterales infections in COVID-19 patients. Infect. Dis..

[19] Onorato L., Sarnelli B., D’Agostino F., Signoriello G., Trama U., D’Argenzio A., Montemurro M.V., Coppola N. (2022). Epidemiological, Clinical and Microbiological Characteristics of Patients with Bloodstream Infections Due to Carbapenem-Resistant, *K. pneumoniae* in Southern Italy: A Multicentre Study. Antibiotics.

[20] Falcone M., Bassetti M., Tiseo G., Giordano C., Nencini E., Russo A., Graziano E., Tagliaferri E., Leonildi A., Barnini S. (2020). Time to appropriate antibiotic therapy is a predictor of outcome in patients with bloodstream infection caused by KPC-producing *Klebsiella pneumoniae*. Crit. Care.

[21] Falcone M., Tiseo G., Galfo V., Giordano C., Leonildi A., Marciano E., De Simone P., Biancofiore G., Boggi U., Barnini S. (2021). Bloodstream infections in patients with rectal colonization by *Klebsiella pneumoniae* producing different type of carbapenemases: A prospective, cohort study (CHIMERA study). Clin. Microbiol. Infect..

[22] Ahmadi M., Ranjbar R., Behzadi P., Mohammadian T. (2022). Virulence factors, antibiotic resistance patterns, and molecular types of clinical isolates of *Klebsiella pneumoniae*. Expert Rev. Anti Infect. Ther..

[23] Mendes G., Ramalho J.F., Duarte A., Pedrosa A., Silva A.C., Méndez L., Caneiras C. (2022). First Outbreak of NDM-1-Producing *Klebsiella pneumoniae* ST11 in a Portuguese Hospital Centre during the COVID-19 Pandemic. Microorganisms.

[24] Ripa M., Galli L., Poli A., Oltolini C., Spagnuolo V., Mastrangelo A., Muccini C., Monti G., De Luca G., Landoni G. (2021). Secondary infections in patients hospitalized with COVID-19: Incidence and predictive factors. Clin. Microbiol. Infect.

[25] Seo H., Kim H.J., Kim M.J., Chong Y.P., Kim S.H., Lee S.O., Choi S.H., Kim Y.S., Woo J.H., Jung J. (2021). Comparison of clinical outcomes of patients infected with KPC- and NDM-producing Enterobacterales: A retrospective cohort study. Clin. Microbiol. Infect..

[26] Bavaro D.F., Belati A., Diella L., Stufano M., Romanelli F., Scalone L., Stolfa S., Ronga L., Maurmo L., Dell’Aera M. (2021). Cefiderocol-Based Combination Therapy for “Difficult-to-Treat” Gram-Negative Severe Infections: Real-Life Case Series and Future Perspectives. Antibiotics.

[27] Karaiskos I., Lagou S., Pontikis K., Rapti V., Poulakou G. (2019). The “Old” and the “New” Antibiotics for MDR Gram-Negative Pathogens: For Whom, When, and How. Front. Public Health.

[28] Bavaro D.F., Romanelli F., Stolfa S., Belati A., Diella L., Ronga L., Fico C., Monno L., Mosca A., Saracino A. (2021). Recurrent neurosurgical site infection by extensively drug-resistant *P. aeruginosa* treated with cefiderocol: A case report and literature review. Infect. Dis..

[29] Guilhaumou R., Benaboud S., Bennis Y., Dahyot-Fizelier C., Dailly E., Gandia P., Goutelle S., Lefeuvre S., Mongardon N., Roger C. (2019). Optimization of the treatment with beta-lactam antibiotics in critically ill patients-guidelines from the French Society of Pharmacology and Therapeutics (Société Française de Pharmacologie et Thérapeutique-SFPT) and the French Society of Anaesthesia and Intensive Care Medicine (Société Française d’Anesthésie et Réanimation-SFAR). Crit. Care.

[30] Delattre I.K., Hites M., Laterre P.F., Dugernier T., Spapen H., Wallemacq P.E., Jacobs F., Taccone F.S. (2020). What is the optimal loading dose of broad-spectrum β-lactam antibiotics in septic patients? Results from pharmacokinetic simulation modelling. Int. J. Antimicrob. Agents.

[31] Rhodes N.J., MacVane S.H., Kuti J.L., Scheetz M.H. (2014). Impact of loading doses on the time to adequate predicted beta-lactam concentrations in prolonged and continuous infusion dosing schemes. Clin. Infect. Dis..

